# Prognostic significance of p53, Sox11, and Pax5 co-expression in mantle cell lymphoma

**DOI:** 10.1038/s41598-021-91433-7

**Published:** 2021-06-07

**Authors:** Caixia Jing, Yuhuan Zheng, Yu Feng, Xia Cao, Caigang Xu

**Affiliations:** 1 Department of Hematology/Hematology Research Laboratory, West China Hospital, Sichuan University, #37 Guo Xue Xiang Street, Chengdu, 610041 China; 2 Department of Hematology, Affiliated Hospital of Southwest Medical University, Luzhou, China; 3 State Key Laboratory of Biotherapy and Cancer Centre, West China Hospital, Sichuan University, Chengdu, China

**Keywords:** Cancer, Biomarkers, Medical research

## Abstract

Mantle cell lymphoma (MCL) is a relatively rare subtype of non-Hodgkin’s lymphoma. To identify molecular biomarkers in MCL, we performed immunohistochemistry tissue arrays using biopsies from 64 MCL patients diagnosed in West China Hospital from 2012 to 2016. *TP53* mutation status in those patients was also examined by sequencing. The sequencing results showed *TP53* mutations were highly heterogeneous in MCL. We identified four novel *TP53* mutations in MCL: P151R, G199R, V218E, and G325R. The MCL patients with *TP53* mutations had inferior progression-free survival (PFS, p = 0.002) and overall survival (OS, p = 0.011). Tissue array results showed the expression of p53, Sox11, or Pax5 alone did not correlate with the patient PFS and OS. However, the MCL patients with triple-positive expression of p53/Sox11/Pax5 had inferior PFS (p = 0.008) and OS (p = 0.002). Such risk stratification was independent to the mantle cell lymphoma international prognostic index (MIPI), Ki-67 value, and *TP53* mutation status of the patients. The triple-positive patients might represent a subtype of high-risk MCL. Our findings might indicate a novel way to stratify MCL and predict patients’ prognosis.

## Introduction

Mantle cell lymphoma (MCL) is a mature B-cell non-Hodgkin’s lymphoma (NHL) and accounts for about 6% of all NHL cases^1^. Based on an epidemiology study in the United States, the incidence rate of MCL was about 0.5 to 0.6 per 100,000 persons, and the rate has increased 2–3 times in the past decades^2^. The primary genetic event of MCL is translocation t(11;14)(q13;q32), which results in cyclin D1 overexpression. However, the dysregulation of cyclin D1 alone may not be sufficient to trigger MCL pathogenesis and aggressiveness^3^. There are many heterogeneous secondary genetic alterations in MCL. For examples, mutations in *INK4A*^4^, *ATM*^5^*, CDK4*^6^*,* and *TP53*^7^ genes are commonly seen in different MCL patients. Those alterations further target the cell signaling pathways, such as cell cycle progression, DNA damage response, and cell survival regulation, thus promoting tumor malignancy^3,8^. MCL remains an incurable lymphoma without any standardized first-line treatment strategy. In addition, MCL is a highly heterogeneous disease; some MCL is aggressive with a median survival of only 3 years while some MCL is indolent that patients can be observed for a period of time before initiating their first treatment^9,10^. Identification of prognostic biomarkers in MCL provides insight for MCL research, not only for risk stratification and personalized treatment optimization but also for demonstrations of MCL biology. In this study, we examined p53, Sox11, and Pax5 expressions in MCL prognosis. Our data suggested a novel risk stratification of MCL based on immunohistochemistry (IHC) analysis of target antigens’ expression.

## Results

### *TP53* mutations in mantle cell lymphoma

**Figure 1:**
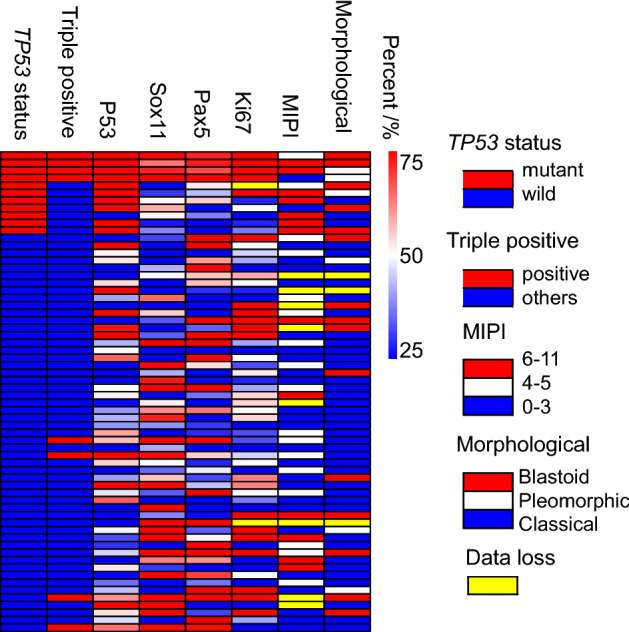
Heatmap of mantle cell lymphoma patients’ characteristics.

**Figure 2:**
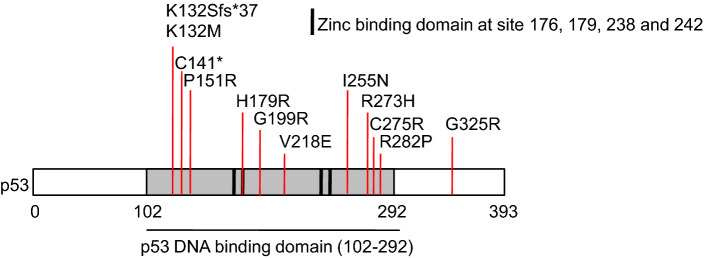
TP53 mutations in West China Hospital cohort of mantle cell lymphoma.

To investigate *TP53* mutations in MCL, we used 64 patient tissue biopsies for target gene sequencing. The patients’ characteristics at diagnosis are summarized in Table 1 and Fig. 1. We found 12 *TP53* mutations in 11 MCL patients (17.19%), and one patient had 2 *TP53* mutations (Supplementary Fig. S1, Fig. 2). There were 10 missense mutations (83.33%), one nonsense mutation (8.33%), and one frame-shift mutation (8.33%). Five *TP53* mutations occurred in exon 5, three mutations in exon 8, two mutations in exon 6, and one mutation in exon 7 and 9, respectively. Eleven mutations resulted in amino acid alteration in the p53 DNA binding domain (91.67%), while one resulted in amino acid alteration at the C terminal of p53.

**Table 1:** Mantle cell lymphoma patients’ characteristics.

	Total Pt # 64
Median age (range)	61 (36–82)
Male, no. (%)	52 (81.3)
HB, g/L (mean ± SD)	122.62 ± 23.82
Median WBC, 10^9^/L (range)	7.25 (2.97–70.21)
Median PLT, 10^9^/L (range)	137 (38–407)
Median LDH, IU/L (range)	220 (136–827)
MIPI^high^, no. (%)	14/56 (21.9)
Median Ki-67, % (range)	25 (4–80)
CD5-, no. (%)	10 (15.6)
**Morphological variants**	
Classical, no. (%)	40/61 (65.6)
Pleomorphic, no. (%)	6/61 (9.8)
Blastoid variant, No. (%)	15/61 (24.6)
**Treatment**	
CHOP (%)	18 (28.1)
R-CHOP (%)	40 (62.5)
R-CHOP/DHAP (%)	3 (4.7)
R-CHOP/R maintenance (%)	1 (1.6)
R-CHOP/DHAP/R maintenance (%)	1 (1.6)
R-CHOP/DHAP/ASCT (%)	1 (1.6)

*HB* hemoglobin, *SD* standard deviation, *WBC* white blood cell, *LDH* lactate dehydrogenase, *MIPI* mantle cell lymphoma international prognostic index.

We reviewed literatures and databases for identified *TP53* mutations in MCL (Supplementary Table S1). In 18 independent studies of a total of 956 MCL patients, 151 patients had *TP53* mutations (15.8%). *TP53* mutations in MCL exhibited high heterogeneity, located at more than 70 different sites in the *TP53* gene. Even the most frequently mutated amino acid residue of p53, R248, occurred only in 13 MCL patients (8.61% in all 151 *TP53* mutated MCL patients). In addition, we didn’t found significant difference between expression of Sox11 and Pax5 in MCL patients with mutant *TP53* and non-mutant *TP53*.

### p53, Sox11, and Pax5 triple-positive mantle cell lymphoma patients have adverse prognosis

**Figure 3:**
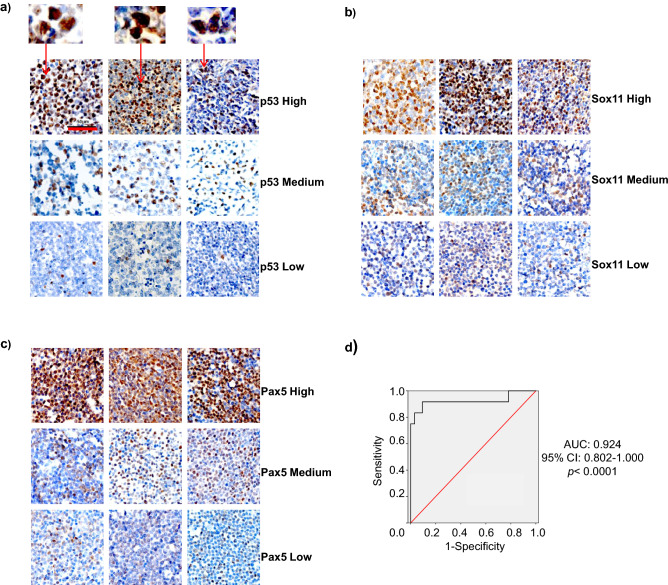
Tissue array of mantle cell lymphoma. Immunohistochemistry tissue arrays of MCL patients’ samples for (**a**) p53, (**b**) Sox11, and (**c**) Pax5. Nine representative out of 64 of each protein stainings were presented. The red arrow represented p53 positive cells in the MCL cells and zoomed images were above; (**d**) ROC curve analysis of p53 immunohistochemistry as a parameter to discriminate between *TP53* mutated and non-mutated samples.

**Figure 4:**
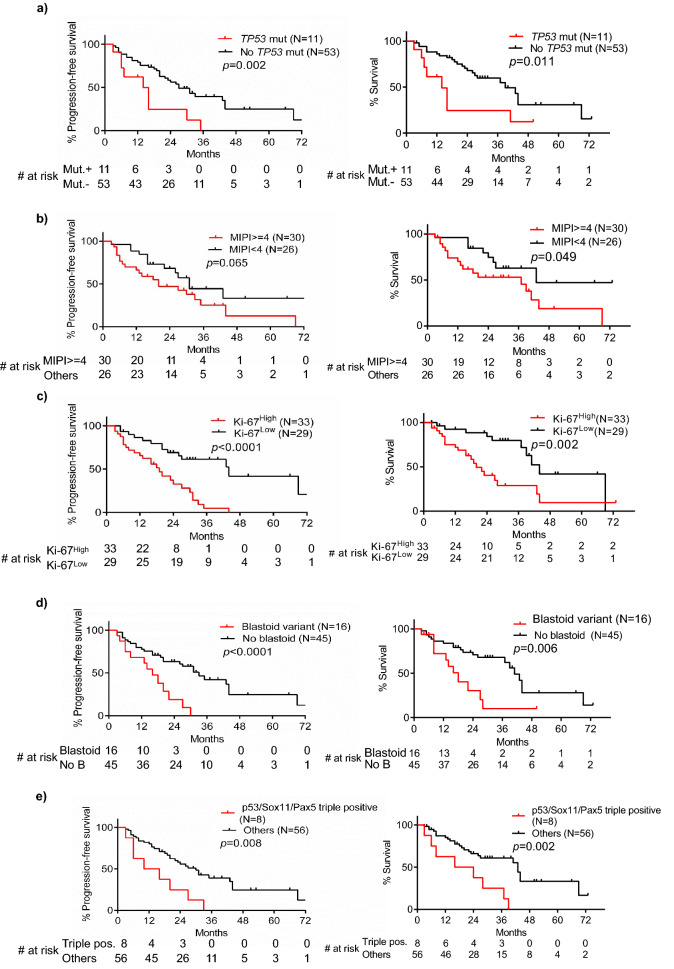
Risk factors of mantle cell lymphoma. (**a**) of 64 MCL patients, the median PFS was 14 versus 27 months (hazard ratio 2.657, 95% CI of ratio 1.595–12.78, *p* = 0.002) and the median OS was 14 versus 39 months (hazard ratio 2.663, 95% CI of ratio 1.408–13.09, *p* = 0.011) for *TP53*^mut+^ versus the others; (**b**) the median PFS was 20 versus 31 months (hazard ratio 1.791, 95% CI of ratio 0.9221–3.544, *p* = 0.065) and the median OS was 37 versus 43 months (hazard ratio 2.186, 95% CI of ratio 1.014–4.739, *p* = 0.049) for MIPI ≥ 4 patients versus the others; (**c**) the median PFS was 19 versus 44 months (hazard ratio 3.19, 95% CI of ratio 2.015–7.286, *p* < 0.0001) and the median OS was 20 versus 44 months (hazard ratio 2.934, 95% CI of ratio 1.545–6.441, *p* = 0.002) for Ki-67^high^ patients versus the others; (**d**) the median PFS was 16 versus 33 months (hazard ratio 2.816, 95% CI of ratio 2.007–11.6, *p* < 0.0001) and the median OS was 18 versus 41 months (hazard ratio 2.616, 95% CI of ratio 1.514–9.706, *p* = 0.006) for patients with blastoid variant cells versus the others. Three patients’ blastoid variant information was not available. (**e**) The median PFS was 13 versus 30 months (hazard ratio 2.794, 95% CI of ratio 1.661–15.9, *p* = 0.008) and the median OS was 20 versus 43 months (hazard ratio 3.210, 95% CI of ratio 2.067–22.26, *p* = 0.002) for triple-positive patients versus the others.

As described below, IHC tissue arrays of 64 MCL patient’s biopsy samples had been performed and representative staining was shown in Fig. 3a–c. The percentage of MCL cells in the biopsy samples and p53 positive cells in the MCL cells was shown in Supplementary Table S2. Most (84.4%) of biopsy samples contained at least 70% MCL cells. As shown in Table 2, 3 out of 64 samples had high p53 expression, 3 had medium p53 expression, and 16 had low p53 expression. 4 samples had high Sox11 expression, 27 had medium expression, and 25 had low expression. 11 samples had high Pax5 expression, 22 had medium expression, and 28 had low expression. Next, we quantified our result. The H-scores of each protein expression were calculated as described in Methods and Materials, as well as in Supplementary Fig. S2. The ROC curve showed that high p53 expression (large H-score in p53 IHC) predicted *TP53* gene mutation well in the patient (Fig. 3d; cutoff H-score value 62, 95% confidence interval (CI) 0.802–1.000, *p* < 0.0001). The specificity and sensitivity of p53 IHC in predicting *TP53* gene mutation were 89.1% and 91.7%, respectively. The MCL patients with *TP53* mutation (17.19%) had inferior PFS and OS compared with other patients (Fig. 4a). In addition, the intermediate/high value of Mantle Cell Lymphoma International Prognostic Index (MIPI) (MIPI ≥ 4), high Ki-67 value (Ki-67 > 30%), and blastoid variant were each correlated with inferior PFS and OS of MCL (Fig. 4b–d, respectively).

**Table 2:** Result of immunohistochemistry staining.

	Negative (%)	Low positive (%)	Positive (%)	High positive (%)	Total positive (%)
p53	42 (65.6)	16 (25)	3 (4.7)	3 (4.7)	22 (34.4)
Sox11	8 (12.5)	25 (39.1)	27 (42.2)	4 (6.3)	56 (87.5)
Pax5	3 (4.7)	28 (43.8)	22 (34.4)	11 (17.2)	61 (95.3)

Our findings suggested that Sox11 and Pax5 alone do not correlate with MCL PFS and OS (data not shown). In our study cohort, the MCL patients with high p53 expression seemed to have inferior OS, but not statistically significant (Supplementary Fig. S3). Those three proteins, Sox11, Pax5 and p53, randomly expressed in each MCL patients without any obvious co-expressions (Supplementary Fig. S4a). However, further analysis suggested that the MCL patients with triple-positive (or triple-high) expression (H-scores p53 > 40, Sox11 > 100, and Pax5 > 100) of target proteins had significantly inferior PFS and OS (Fig. 4e). Double-positive expression of either 2 genes did not correlate with prognosis (data not shown). It was notable that the clinical features of the triple-positive patients and the rest of patients had no statistical difference (Table 3). We summarized *TP53* mutation status in triple positive patients versus the others (Supplementary Table S3). We didn’t identify any correlations. Some of the features such as MIPI^high^, Ki-67^high^, and Blastoid variant were correlated with MCL prognosis, but those features had no enrichment in triple-positive patients. Therefore, the triple-positive stratification of MCL might be independent to those known prognostic factors. Multivariate Cox-regression analysis confirmed that triple-positive stratification was independent to prognostic factors such as *TP53* mutation status, Ki-67 value and MIPI (Table 4). In addition, evidence suggested that Sox11 regulated Pax5 expression in MCL^11^. However, our IHC array showed that a significant amount of MCL patients didn’t have Sox11 and Pax5 co-expression (Supplementary Fig. S4b).

**Table 3:** Comparison of triple-positive patients with the other patients.

Variable	Triple positive	Others	*p* value
No. (%)	8 (12.5)	56 (87.5)	
Median age (range)	62.5 (47–72)	61 (36–82)	0.911
Male, no. (%)	7 (87.5)	45 (80.4)	> 0.99
*TP53* mutated, no. (%)	4 (50)	7 (12.5)	0.024
Median p53 H-score (range)	89.55 (42.38–204.56)	34.5 (8.89–214.61)	0.001
Median Sox11 H-score (range)	145.88 (120.13–162.79)	80.6 (16.9–241.72)	0.007
Median Pax5 H-score (range)	160.84 (114.4–250.36)	84.12 (28.84–258.86)	0.012
HB, g/L ( mean ± SD)	113.83 ± 21.11	123.82 ± 24.14	0.341
Median WBC, 10^9^/L (range)	8.54 (5.3–70.21)	6.97 (2.97–43.94)	0.178
Median PLT, 10^9^/L (range)	194 (128–332)	136 (38–407)	0.077
Median LDH, IU/L (range)	328 (151–827)	218 (136–424)	0.515
MIPI^high^, no. (%)	2 (28.6)	12 (24.5)	> 0.99
Blastoid variant, no. (%)	3 (37.5)	12 (22.6)	0.393
Median Ki-67, % (range)	60 (10–80)	25 (4–80)	0.107
CD5-/ ± , no. (%)	4 (50)	16 (28.6)	0.244
**Treatment**			0.845
CHOP (%)	3 (37.5)	15 (26.8)	
R-CHOP (%)	5 (62.5)	36 (64.3)	
R-CHOP/DHAP (%)	0 (0)	5 (8.9)

**Table 4:** Cox-regression analysis of triple-positive patients.

Variable	Univariate			Multivariate		
	HR	95% CI	*p*	HR	95% CI	*p*
**Progression-free survival**						
Triple positive	2.752	1.253–6.043	0.012	2.476	0.985–6.224	0.054
TP53 mut	3.045	1.459–6.352	0.003	1.821	0.759–4.368	0.179
Ki-67 > 30%	3.253	1.662–6.366	0.001	1.525	0.661–3.517	0.322
MIPI (continuous)	1.226	1.043–1.441	0.014	1.107	0.915–1.339	0.296
Blastoid variant	3.839	1.842–8.001	< 0.0001	2.962	1.249–7.023	0.014
**Overall survival**						
Triple positive	3.387	1.481–7.745	0.004	3.816	1.475–9.875	0.006
TP53 mut	2.729	1.210–6.152	0.016	1.580	0.603–4.137	0.352
Ki-67 > 30%	2.603	1.242–5.458	0.011	1.085	0.42–2.805	0.866
MIPI (continuous)	1.255	1.041–1.513	0.017	1.159	0.916–1.468	0.219
Blastoid variant	4.819	2.092–11.099	< 0.0001	4.125	1.517–11.216	0.005

## Discussion

According to the literatures and databases, four *TP53* mutations detected in our patients—P151R, G199R, V218E, and G325R—had never been reported in MCL. Among those mutations, P151R, G199R, and V218E were located in the p53 DNA binding domain. Thus, those mutations might affect p53 function and promote oncogenic transformation. G325R mutation had been reported in breast cancer with uncertain clinical significance^12^. This mutation located in the oligomerization domain of p53 and therefore might affect p53 tetramerization^13^. Those mutations expanded the mutation spectrum of *TP53* in MCL. Some researchers guessed that the controversial relationship between Sox11 expression and prognosis of MCL might be influenced by *TP53* mutant status^14,15^, but no correlation was found between Sox11 expression and *TP53* mutant status in our study.

MIPI and Ki-67 have been used clinically for MCL-risk stratification and prognosis prediction^16^. Several other factors, such as patients’ beta-2 microglobulin level, *TP53* mutation status, expressions of Sox11, SOC3, Myc, or Pax5 might also harbor prognostic values in MCL^11,17–20^. In our study, the prognostic significance of MIPI, Ki-67, and *TP53* mutation status in MCL patients was in agreement with previous research results. Those findings in our patient cohort provided confidence of our sample collection. We also investigated prognostic significance of p53, Sox11, and Pax5 expression in MCL patients. Previous studies on those 3 factors were controversial. Some studies showed that each of these 3 factors was correlated with MCL prognosis, but others had negative findings^20–22^. In particular, according to the revised 4th edition of “WHO Classification of Tumours of Haematopoietic and Lymphoid Tissues”, the non-nodal MCL, which was indolent, had negative Sox11 expression, therefore Sox11^high^ MCL patients might have inferior OS in a cohort with both non-nodal and classic MCL patients^9,23,24^. However, Nygren L et al. presented an opposite finding that most indolent MCLs were Sox11 positive^14^. More recently, an European immunohistochemistry study of MCL biopsies suggested that Sox11^low^ or p53^high^ patients had inferior OS^15^. By analyzing peripheral blood, adenopathy, and Sox11 expression et al. synthetically, there was no non-nodal type of MCL in our 64-patient cohort. Based on our results in MCL IHC, neither p53, Sox11, nor Pax5 expression alone was correlated with the patient’s PFS or OS. The p53^high^ MCL patients had slightly worse OS than the p53^low^ patients but had no statistical significance. If *TP53*^mut^ patients were excluded in comparison (most *TP53*^mut^ patients had high p53 expression in IHC), the OS of p53^high^ patients had no difference from other patients. According to previous publications, whether the p53 protein level correlated with MCL OS is still controversial. Several publications suggested that the expression of p53 associated with MCL OS^14,15,25^. However, Greiner et al. assessed p53 expression at the protein level in MCL patients, but only reported correlation between TP53 mutations and the patients’ OS^7^. Stefancikova and Zlamalikova et al. also were unable to find relevance between expression of p53 protein and MCL prognosis^26,27^. Thus, the single antigen expression of p53, Sox11, or Pax5 might not be sufficient to predict MCL prognosis. Of notice, most studies addressed the prognostic value of p53 expression in MCL using p53 protein instead of mRNA. P53 had post-transcriptional level regulations, such as translation regulation by internal ribosomal entry site (IRES)^28^ and protein stability regulation^29^. Thus, there might be inconsistence between p53 expressions at the mRNA level and the protein level.

Our result suggested that the MCL patients with triple-positive expression of p53/Sox11/Pax5 had inferior OS. Such correlation did not stand on any identified prognostic factors of MCL, such as MIPI, Ki-67, or *TP53* mutation status. Why triple-positive MCL had inferior OS were not clear. Large cohort study was necessary to draw a confirmative conclusion. Previous studies demonstrated that Sox11 played an oncogenic role in MCL by modulating cell cycle, apoptosis, and cell differentiation. Pax5 was regulated by Sox11 at the transcriptional level^11^. On the other hand, the function of p53 in MCL, in particular its interplay with other MCL oncogenic factors, was not well demonstrated. Yang P et al. suggested that aberrant p53 might promote cell cycle dysregulation in MCL^30^. Teo et al. mentioned that the TP53−/− MCL cell line had altered chemo-sensitivity^22^. Based on the above findings, overexpression of p53, Sox11, or Pax5 promotes MCL malignancy. Thus, it was reasonable to predict that p53/Sox11/Pax5 triple-positive MCL might represent a highly aggressive tumor subtype with inferior prognosis. In our data, the inconsistence of Sox11 and Pax5 co-expression in MCL suggested that, at least in some MCL, the Pax5 expression might be controlled by factors other than Sox11. Furthermore, Teo et al. showed that Pax−/− MCL had increased cell proliferation in vitro and more aggressive tumor behavior in vivo^22^. Teo’s study also indicated a Pax5-initiated regulation on p53. Therefore, in some MCL, Sox11 and Pax5 regulatory hierarchy and p53 regulation might not be canonical as we had anticipated.

It should be noted that there are several limitations of our study. (1) Small patient number; (2) heterogeneous treatment regimen; (3) lacks the study of mechanisms. Therefore, additional prospective studies with lager cohorts and homogenous treatment regimen are needed to verify our results. The potential pathophysiological mechanisms of these results deserve further investigation.

To summarize, the MCL patients with triple positive p53/Sox11/Pax5 expression had inferior disease. Such MCL risk stratification was independent to MIPI, Ki-67, or *TP53* mutation status. The biology underneath such observation is still largely unknown. Future validation and demonstration of this MCL risk stratification might provide new perspective on MCL pathology.

## Materials and methods

### Patient samples

Formalin-fixed, paraffin-embedded tissue biopsies from 64 MCL patients were included in this study. The patients were diagnosed in West China Hospital, Sichuan University from 2012 to 2016. The MCL diagnostic standards were based on WHO guidelines^31^. All patients had cyclin D1 overexpression in tissue biopsies and had t(11;14)(q13;q32) detected by fluorescence in situ hybridization (FISH). The patients received R-CHOP/CHOP (Rituximab, Cyclophosphamide, Doxorubicin, Oncovin, Prednisone), or R-DHAP (Dexamethasone, Cytarabine, Cisplatin) treatment, Among them, two patients received rituximab maintenance, one patient received autologous stem cell transplantation (ASCT). Of the 64 MCL patients, the median follow-up time was 23 months with a range of 3–73 months. The Ethical Committee of Sichuan University approved this study and waived informed consent. All methods were performed in accordance with the relevant guidelines and regulations.

### Assessment of *TP53* mutations in patient samples

Genomic DNA was isolated from paraffin-embedded tissues using QIAmp DNA FFPE Tissue Kit (Qiagen) following the manufacturer’s instruction. *TP53* exons 2–11, including the intron–exon boundaries, were sequenced by Sanger sequencing. Supplementary Table S4 given the primer sequences. The result was compared with the International Agency for Research on Cancer (IARC) *TP53* database, the Catalogue of Somatic Mutations in Cancer (COSMIC) database, and the UMD *TP53* mutation database to identify novel mutations. Wild-type *TP53* sequence was acquired from NCBI.

### Immunohistochemical analysis of tissue array and results quantification

Paraffin-embedded tissue biopsies from the patients were sectionized and used for IHC analysis as described earlier^32^. The primary antibody against human p53 was ordered from Thermo Fisher Inc (MA5-12557). The primary antibody against Sox11 was ordered from ZSBIO (ZM-0366), CHINA. The primary antibodies against Pax5 was ordered from Abcam (ab109443). All antibodies were diluted with phosphate buffer solution (PH 7.3) and had been verified for immunohistochemistry staining. Percentage of MCL cells in the biopsy samples and positive nuclei in the MCL cells were determined by 2 pathologists under microscopy independently. The results were in good concordance. For the different results, 2 pathologists determined the end results together. According to a previous study^15^ and appropriate adjustments, proteins were defined as negative (0% positive MCL cells), low (1–29% positive MCL cells), intermediate (30–49% positive MCL cells) or high (≥ 50% positive MCL cells). The results of staining were captured by microscopy using AX10 Imager A1/Cam HRC and Zeiss at × 40 magnification, and were quantified by ImageJ with the IHC profiler plugin as described earlier^33^. The results were rendered as a pixel intensity histogram to demonstrate the percentage contribution of high positive, positive, low positive, and negative. The scoring assignment was performed via H-scoring. The formula was as follows: H-score = (% of high positive × 3) + (% of positive × 2) + (% of low positive × 1). The H-score had a range from 0 to 300. Based on a previous publication^34^, with some modifications, we defined H-score > 100 as positive for Sox11 and Pax5 and H-score > 40 as positive for p53.

### Statistical analysis

Statistical analyses were performed using SPSS Statistics (Version 19) and GraphPad. Comparisons of two groups were analyzed by Student’s *t*-test. Specifically, the Mann–Whitney test was used for quantitative variables and χ^2^ or Fisher’s exact test was used for categorical variables. The receiver operating characteristic (ROC) curve was used to analyze the level of p53 IHC expression in predicting *TP53* mutation. Progression-free survival (PFS) and overall survival (OS) were estimated and compared using the Kaplan–Meier method (Log-rank test). Cox proportional hazard models (univariate and multivariate) were used to identify independent prognostic factors. A *p* < 0.05 was considered statistically significant.

## Supplementary Information

Supplementary Figures.

Supplementary Tables.

## Data Availability

The authors declare that the main data supporting the results of the current study are available from the corresponding authors on reasonable request.
