# Supplementary material for: Prognostic significance of p53, Sox11, and Pax5 co-expression in mantle cell lymphoma
Source: Sci Rep. 2021 Jun 7;11:11896. doi: 10.1038/s41598-021-91433-7 (PMC8185106; doi:10.1038/s41598-021-91433-7)
Supplement: Supplementary file 1 — Supplementary Figures. [file 41598_2021_91433_MOESM1_ESM.pdf]

Title Page

Prognostic Significance of p53, Sox11, and Pax5 Co-expression in Mantle Cell Lymphoma

Caixia Jing[1,2]*, Yuhuan Zheng[1,3]*, Yu Feng[1], Xia Cao[1], Caigang Xu[1]

[1]Department of Hematology/Hematology Research Laboratory, West China Hospital, Sichuan University, Chengdu, China

[2]Department of Hematology, Affiliated Hospital of Southwest Medical University, Luzhou, China

[3]State Key Laboratory of Biotherapy and Cancer Centre, West China Hospital, Sichuan University, Chengdu, China

* C.J. and Y.Z. contributed equally to this manuscript

Corresponding author: **Caigang Xu**, M.D., Department of Hematology, West China Hospital, Sichuan University;#37 Guo Xue Xiang Street, Chengdu, China 610041;

E-mail: xucaigang@wchscu.cn; ORCID: 0000-0003-0549-4065

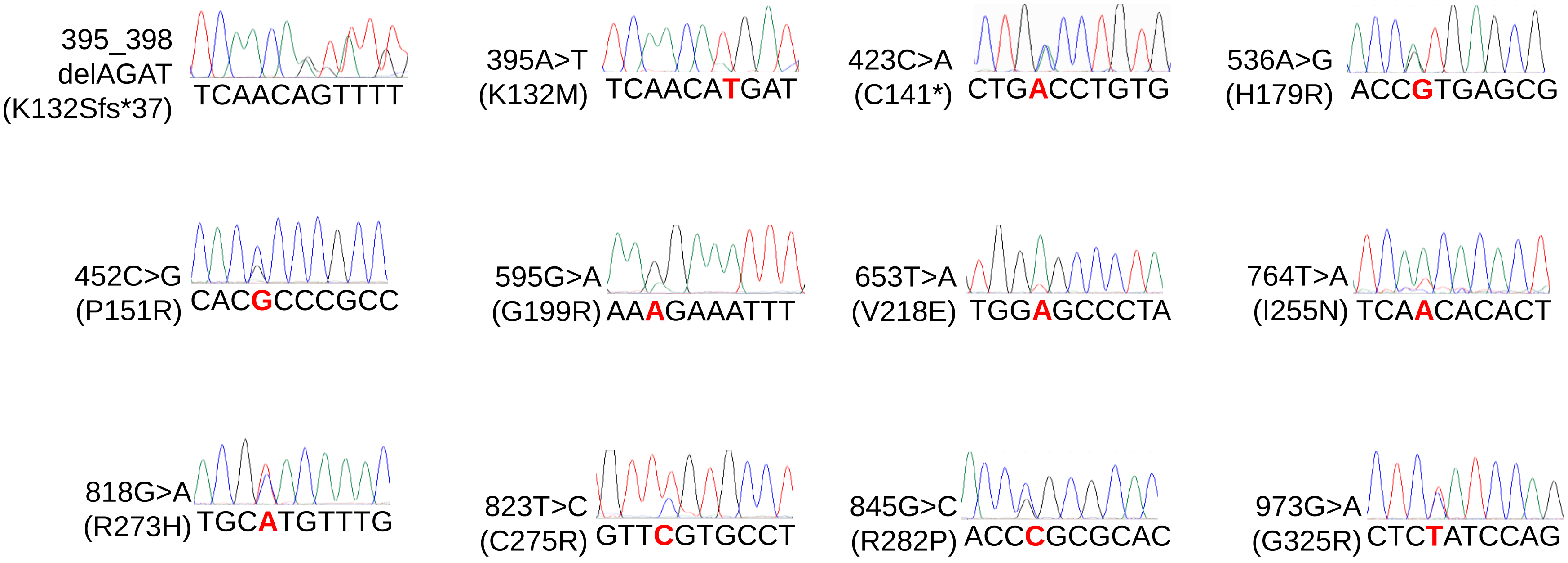

Supplementary Fig. S1 *TP53* mutations in West China Hospital cohort of mantle cell lymphoma

a)

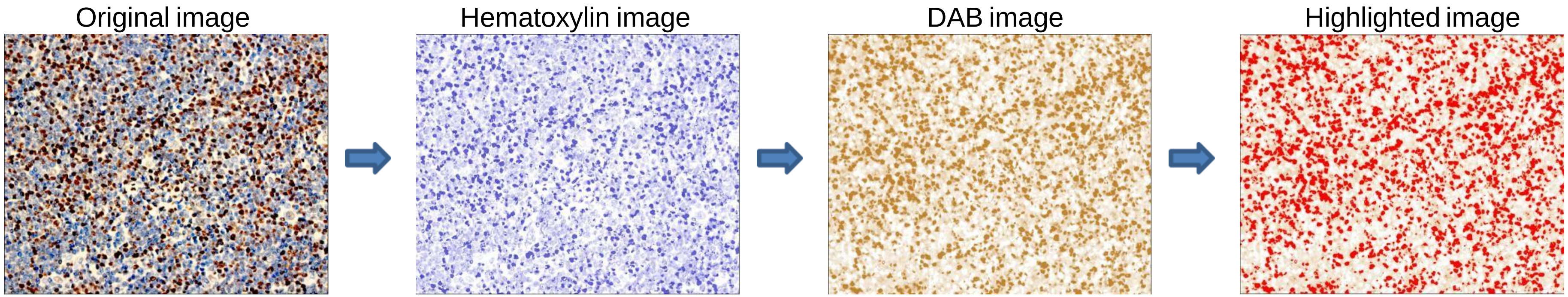

b)

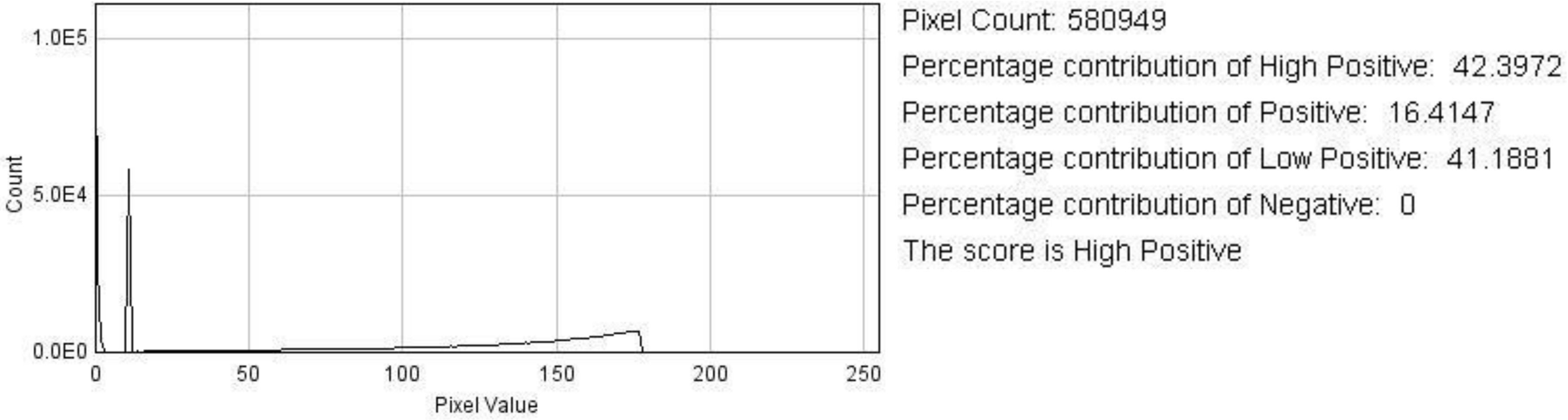

To quantify immunohistochemistry staining, each original staining image, as shown in representative in **a)** from left to right, was color deconvoluted to isolate the hematoxylin and DAB brown staining. The positive staining on the DAB image were highlighted as red spots; **b)** The highlighted image was used for result quantification. Pixel values had a range from 0-255, and were divided into four subcategories: high positive (0–60), positive (61–120), low positive (121–180), and negative (181–255).

Supplementary Fig. S2 Representative immunohistochemistry result quantification

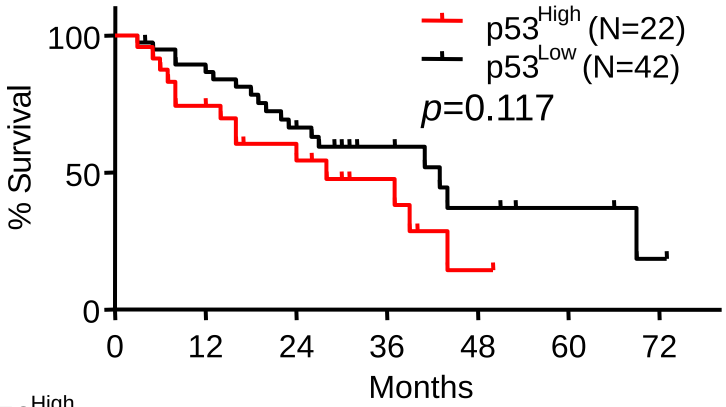

# at risk		0	12	24	36	48	60	72
	$p53^{High}$	24	17	10	6	2	1	1
	$p53^{Low}$	40	33	21	11	6	4	2

of 64 MCL patients, the median OS was 28 versus 43 months (hazard ratio 1.845, 95% CI of ratio.8577-3.967, *p*=0.117) for $p53^{high}$ patients versus the others.

Supplementary Fig. S3 The expression of p53 and MCL patient overall survival

a)

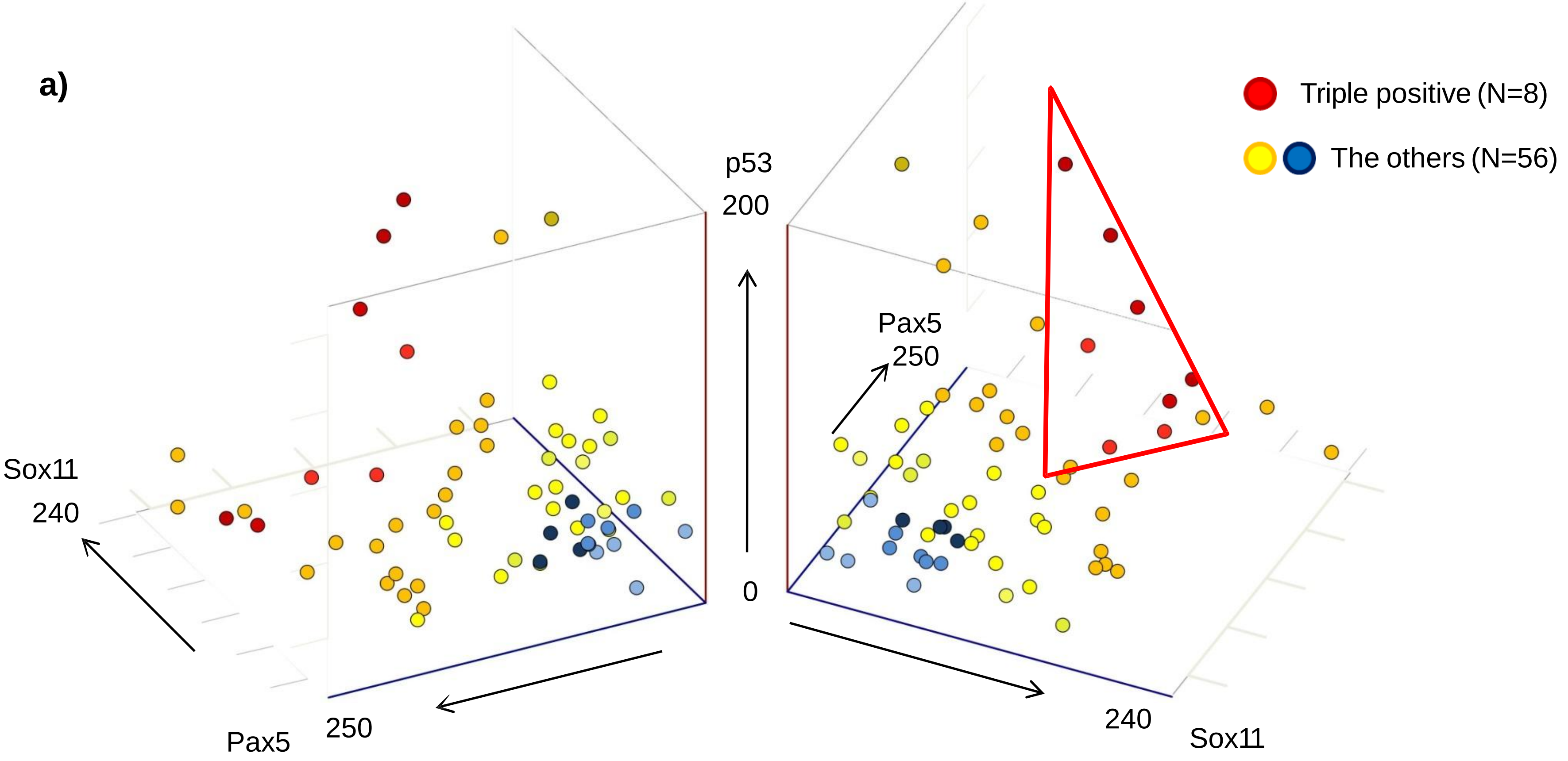

b)

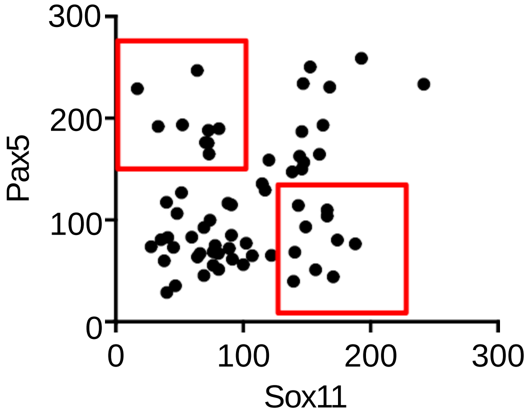

a) 3D graph showing the expression of p53, Sox11, and Pax5 in each MCL patient. The H-score range of p53 was 0-200, the range of Pax5 was 0-250, and the range of Sox11 was 0-240. The red triangle emphasize the triple-positive (red dot; H-scores p53>40, Sox11>100, and Pax5>100); **b)** expression of Sox11 and Pax5 for each MCL patient. The red square represents patients without co-expression.

Supplementary Fig. S4 The expression of p53, Sox11, and Pax5 in each MCL patient
