# Supplementary material for: Prognostic significance of p53, Sox11, and Pax5 co-expression in mantle cell lymphoma
Source: Sci Rep. 2021 Jun 7;11:11896. doi: 10.1038/s41598-021-91433-7 (PMC8185106; doi:10.1038/s41598-021-91433-7)
Supplement: Supplementary file 2 — Supplementary Tables. [file 41598_2021_91433_MOESM2_ESM.docx]

**Title Page**

**Prognostic Significance of p53, Sox11, and Pax5 Co-expression in Mantle Cell Lymphoma**

Caixia Jing^1,2^*, Yuhuan Zheng^1,3^*, Yu Feng^1^, Xia Cao^1^, Caigang Xu^1^

^1^Department of Hematology/Hematology Research Laboratory, West China Hospital, Sichuan University, Chengdu, China

^2^Department of Hematology, Affiliated Hospital of Southwest Medical University, Luzhou, China

^3^State Key Laboratory of Biotherapy and Cancer Centre, West China Hospital, Sichuan University, Chengdu, China

* C.J. and Y.Z. contributed equally to this manuscript

Corresponding author: **Caigang Xu**, M.D., Department of Hematology, West China Hospital, Sichuan University;#37 Guo Xue Xiang Street, Chengdu, China 610041; E-mail: [xucaigang@wchscu.cn](mailto:xucaigang@wchscu.cn); ORCID: [0000-0003-0549-4065](javascript:popup_orcidDetail('https://orcid.org','0000-0003-0549-4065');)

| **No.** | **PMID** | **Country** | **Pt # with TP53**  **mutant/Total pt** | **TP53 mutations** | | | | | | | | | | | | | | | | | | | | | | | | | | | | | | | | | | | | | | |
| --- | --- | --- | --- | --- | --- | --- | --- | --- | --- | --- | --- | --- | --- | --- | --- | --- | --- | --- | --- | --- | --- | --- | --- | --- | --- | --- | --- | --- | --- | --- | --- | --- | --- | --- | --- | --- | --- | --- | --- | --- | --- | --- |
| 1 | 29794145 | Denmark | 5/44 |  |  |  |  |  |  |  |  |  |  |  | N131Pfs |  |  |  |  |  |  |  |  |  |  |  |  |  |  |  |  |  |  |  |  |  |  |  |  |  |  |  |
| 2 | 28819011 | Denmark | 21/176 | A39fs | E51* |  |  |  |  |  |  |  |  |  |  | K132E |  |  |  |  |  |  |  |  |  |  |  |  |  |  |  |  | H179R |  |  |  | P191R |  | L194R |  |  | V197E |
|  |  |  |  |  |  |  |  |  |  |  |  |  |  |  |  |  |  |  |  |  |  |  |  |  |  |  |  |  |  |  |  |  | H179Q |  |  |  |  |  |  |  |  |  |
| 3 | 28791403 | Czech  Republic | 13/57 |  |  |  |  |  | R110L |  |  |  |  |  |  |  |  |  | P142Lfs*27 |  |  |  |  |  |  | Y163C |  |  |  |  | R175H |  |  |  |  |  |  |  |  |  |  |  |
| 4 | 24682267 | US | 11/56 |  |  |  | P58fs | A78_P80del |  |  |  |  |  |  |  |  |  |  |  |  |  |  |  |  |  |  |  |  |  |  | R175H(2) |  |  |  |  |  |  |  |  | I195T | R196P |  |
| 5 | 24145436 | Spain | 8/29 |  |  | F54fs |  |  |  |  |  |  | Y126Pfs*7 |  |  |  |  |  |  |  |  |  |  |  |  |  |  |  |  |  |  |  |  | S183* |  |  |  | H193R |  |  |  |  |
|  |  |  |  |  |  |  |  |  |  |  |  |  |  |  |  |  |  |  |  |  |  |  |  |  |  |  |  |  |  |  |  |  |  |  |  |  |  | H193Y |  |  |  |  |
| 6 | 23407552 | Canada | 16/100 |  |  |  |  |  |  | F113Gfs*37 | A119G |  |  | S127P |  |  |  |  |  |  |  |  |  | A159V |  |  | Q165* |  |  |  |  |  | H179Y |  |  |  |  |  |  | I195F | R196* |  |
| 7 | 22315488 | Germany | 8/62 |  |  |  |  |  |  |  |  | T125fs |  |  |  |  | C135Y |  |  |  |  | V157F |  |  |  |  |  |  |  |  |  |  | H179Q |  |  |  |  |  |  |  |  |  |
|  |  |  |  |  |  |  |  |  |  |  |  |  |  |  |  |  |  |  |  |  |  |  |  |  |  |  |  |  |  |  |  |  | H179R |  |  |  |  |  |  |  |  |  |
| 8 | 22057359 | China | 6/32 |  |  |  |  |  |  |  |  |  |  |  |  |  | C135Y |  |  |  |  |  |  |  |  |  |  |  |  |  | R175H |  |  |  |  |  |  |  | L194H |  |  |  |
| 9 | 21720382 | Sweden/  Finland | 16/119 |  |  |  |  |  |  |  |  |  |  |  |  |  |  | C141Y |  |  |  |  |  |  | I162F |  | Q165* |  |  |  | R175H | C176S |  |  |  |  |  |  |  |  |  |  |
|  |  |  |  |  |  |  |  |  |  |  |  |  |  |  |  |  |  |  |  |  |  |  |  |  |  |  |  |  |  |  |  |  |  |  |  |  |  |  |  |  |  |  |
| 10 | 17989721 | UK | 3/22 |  |  |  |  |  |  |  |  |  |  |  |  |  |  |  |  |  |  |  |  |  |  |  |  |  |  |  |  |  |  |  |  |  |  |  |  |  |  |  |
| 11 | 16461462 | US | 15/82 |  |  |  |  |  |  |  |  |  |  |  |  |  |  |  |  |  |  |  | R158_A159del |  |  | Y163H |  | S166* |  |  |  | C176W |  |  |  | A189del_P190V |  |  | L194R |  | R196* |  |
|  |  |  |  |  |  |  |  |  |  |  |  |  |  |  |  |  |  |  |  |  |  |  |  |  |  |  |  |  |  |  |  |  |  |  |  |  |  |  |  |  |  |  |
| 12 | 11793447 | UK | 1/1 |  |  |  |  |  |  |  |  |  |  |  |  |  |  |  |  |  |  |  |  |  |  |  |  |  |  | V173G |  |  |  |  |  |  |  |  |  |  |  |  |
| 13 | 10854221 | Spain | 4/26 |  |  |  |  |  |  |  |  |  |  |  |  |  |  |  |  |  | T155N |  |  |  |  |  |  |  |  |  |  |  |  |  |  |  |  |  |  |  |  |  |
| 14 | 9697882 | Denmark | 1/8 |  |  |  |  |  |  |  |  |  |  |  |  |  |  |  |  |  |  |  |  |  |  |  |  |  |  |  |  |  |  |  |  |  |  |  |  |  |  |  |
| 15 | 8639789 | US | 8/53 |  |  |  |  |  |  |  |  |  |  |  |  |  |  |  |  |  |  |  |  |  |  |  |  |  |  |  |  |  |  |  | D184Efs |  |  |  |  |  |  |  |
| 16 | 8603336 | Italy | 2/2 |  |  |  |  |  |  |  |  |  |  |  |  |  |  |  |  |  |  |  | R158H |  |  |  |  |  | Q167* |  |  |  |  |  |  |  |  |  |  |  |  |  |
| 17 | 7579380 | US | 2/23 |  |  |  |  |  |  |  |  |  |  |  |  | K132R |  |  |  |  |  |  |  |  |  |  |  |  |  |  | R175H | C176G |  |  |  |  |  |  |  |  |  |  |
| 18 |  | China | 11/64 |  |  |  |  |  |  |  |  |  |  |  |  | K132Sfs*37 |  | C141* |  | P151R |  |  |  |  |  |  |  |  |  |  |  |  | H179R |  |  |  |  |  |  |  |  |  |
|  |  |  |  |  |  |  |  |  |  |  |  |  |  |  |  | K132M |  |  |  |  |  |  |  |  |  |  |  |  |  |  |  |  |  |  |  |  |  |  |  |  |  |  |
| **Mutation site** | | | | **A39** | **E51** | **F54** | **P58** | **A78** | **R110** | **F113** | **A119** | **T125** | **Y126** | **S127** | **N131** | **K132** | **C135** | **C141** | **P142** | **P151** | **T155** | **V157** | **R158** | **A159** | **I162** | **Y163** | **Q165** | **S166** | **Q167** | **V173** | **R175** | **C176** | **H179** | **S183** | **D184** | **A189** | **P191** | **H193** | **L194** | **I195** | **R196** | **V197** |
| **Cases (Total MCL Pt.#: 956)** | | | | 1 | 1 | 1 | 1 | 1 | 1 | 1 | 1 | 1 | 1 | 1 | 1 | 4 | 2 | 2 | 1 | 1 | 1 | 1 | 2 | 1 | 1 | 2 | 2 | 1 | 1 | 1 | 6 | 3 | 6 | 1 | 1 | 1 | 1 | 2 | 3 | 2 | 3 | 1 |
|  | | | | | | | | | | | | | | | | | | | | | | | | | | | | | | | | | | | | | | | | | | |
| **No.** | **TP53 mutations** | | | | | | | | | | | | | | | | | | | | | | | | | | | | | | | | | | | | | | | | | |
| 1 |  |  |  |  |  |  |  |  |  |  |  |  | C242Afs*5 |  |  |  |  |  |  |  |  |  | I255N |  |  |  |  |  | R273C |  |  |  |  |  | R282W |  |  |  |  |  |  |  |
| 2 |  |  |  |  |  |  |  |  | Y234N | Y236C |  | N239D | C242W |  |  |  |  | R248W | R249S |  |  | T253fs |  |  |  |  |  |  |  |  |  |  |  |  |  | T284P | E286G |  |  |  |  | T387fs |
|  |  |  |  |  |  |  |  |  |  |  |  |  |  |  |  |  |  | R248Q(3) |  |  |  |  |  |  |  |  |  |  |  |  |  |  |  |  |  |  |  |  |  |  |  |  |
| 3 |  |  |  |  |  |  |  | D228* |  |  | C238S |  |  |  |  |  |  | R248W |  |  |  |  |  |  |  |  |  |  | R273H(2) | C275Y |  |  | R280G |  | R282W |  |  |  |  |  | Q331* |  |
| 4 |  |  |  |  |  |  |  |  |  |  |  |  |  |  | G244C | M246R |  | R248W(2) |  |  |  |  |  | E258D |  |  |  |  |  |  |  |  | R280T |  |  |  |  |  | Q317* |  |  |  |
| 5 |  |  | R213* |  |  |  |  |  |  |  |  |  |  |  |  |  |  |  |  |  |  |  |  |  |  |  |  |  |  | C275F |  |  |  | D281Y |  |  |  |  |  |  | Q331* |  |
|  |  |  |  |  |  |  |  |  |  |  |  |  |  |  |  |  |  |  |  |  |  |  |  |  |  |  |  |  |  |  |  |  |  |  |  |  |  |  |  |  |  |  |
| 6 |  |  |  |  | V216M |  | Y220C |  |  | Y236N |  |  |  |  |  |  |  |  |  | P250L | I251F |  |  |  |  | N268Efs*4 | F270S |  |  |  |  |  |  |  |  |  | E286K |  |  |  |  |  |
| 7 |  |  |  |  |  |  | Y220C |  |  |  |  |  |  | M243V |  |  |  |  |  |  |  |  |  |  |  |  |  |  | R273L |  |  |  |  |  |  |  |  |  |  |  |  |  |
|  |  |  |  |  |  |  |  |  |  |  |  |  |  |  |  |  |  |  |  |  |  |  |  |  |  |  |  |  |  |  |  |  |  |  |  |  |  |  |  |  |  |  |
| 8 |  |  |  |  |  |  |  |  | Y234C |  |  |  | C242Afs*5 |  | G244D |  |  |  |  |  |  |  |  |  |  |  |  |  |  |  |  |  |  |  |  |  |  |  |  |  |  |  |
| 9 |  | Y205D | R213* |  |  |  |  |  |  |  |  |  | C242W |  |  |  |  | R248Q |  |  |  |  |  |  |  |  | F270S | V272M | R273C | C275Y |  |  | R280G | D281N |  |  |  |  |  |  |  |  |
|  |  |  |  |  |  |  |  |  |  |  |  |  |  |  |  |  |  |  |  |  |  |  |  |  |  |  |  |  | R273H |  |  |  |  |  |  |  |  |  |  |  |  |  |
| 10 |  |  |  |  |  |  |  |  |  |  |  |  |  |  |  |  | N247_R249del | R248G |  |  |  |  |  |  |  |  | F270L |  |  |  |  |  |  |  |  |  |  |  |  |  |  |  |
| 11 |  |  |  | S215I |  |  |  |  |  |  |  |  |  |  | G244D |  |  | R248Q(2) |  |  |  |  | I255F |  |  |  |  |  | R273C |  | C277F |  |  |  |  |  |  |  |  |  |  |  |
|  |  |  |  |  |  |  |  |  |  |  |  |  |  |  |  |  |  |  |  |  |  |  |  |  |  |  |  |  | R273H |  | C277* |  |  |  |  |  |  |  |  |  |  |  |
| 12 |  |  |  |  |  |  |  |  |  |  |  |  |  |  |  |  |  |  |  |  |  |  |  |  |  |  |  |  |  |  |  |  |  |  |  |  |  |  |  |  |  |  |
| 13 |  |  |  |  |  |  |  |  |  |  |  |  |  | M243S |  |  |  |  |  |  |  |  |  |  |  |  |  |  | R273H |  |  | P278L |  |  |  |  |  |  |  |  |  |  |
| 14 |  |  |  |  |  |  |  |  |  |  |  |  |  |  |  |  |  |  |  |  |  |  |  |  |  |  |  |  | R273H |  |  |  |  |  |  |  |  |  |  |  |  |  |
| 15 |  |  | R213* |  |  |  |  |  | Y234C |  |  |  |  |  |  |  |  | R248Q(2) |  |  |  |  |  |  | G266V |  |  |  | R273P |  |  | P278T |  |  |  |  |  |  |  |  |  |  |
| 16 |  |  |  |  |  |  |  |  |  |  |  |  |  |  |  |  |  |  |  |  |  |  |  |  |  |  |  |  |  |  |  |  |  |  |  |  |  |  |  |  |  |  |
| 17 |  |  |  |  |  |  |  |  |  |  |  |  |  |  |  |  |  |  |  |  |  |  |  |  |  |  |  |  |  |  |  |  |  |  |  |  |  | R306* |  |  |  |  |
| 18 | G199R |  |  |  |  | V218E |  |  |  |  |  |  |  |  |  |  |  |  |  |  |  |  | I255N |  |  |  |  |  | R273H | C275R |  |  |  |  | R282P |  |  |  |  | G325R |  |  |
|  |  |  |  |  |  |  |  |  |  |  |  |  |  |  |  |  |  |  |  |  |  |  |  |  |  |  |  |  |  |  |  |  |  |  |  |  |  |  |  |  |  |  |
|  | **G199** | **Y205** | **R213** | **S215** | **V216** | **V218** | **Y220** | **D228** | **Y234** | **Y236** | **C238** | **N239** | **C242** | **M243** | **G244** | **M246** | **N247** | **R248** | **R249** | **P250** | **I251** | **T253** | **I255** | **E258** | **G266** | **N268** | **F270** | **V272** | **R273** | **C275** | **C277** | **P278** | **R280** | **D281** | **R282** | **T284** | **E286** | **R306** | **Q317** | **G325** | **Q331** | **T387** |
|  | 1 | 1 | 3 | 1 | 1 | 1 | 2 | 1 | 3 | 2 | 1 | 1 | 4 | 2 | 3 | 1 | 1 | 13 | 1 | 1 | 1 | 1 | 3 | 1 | 1 | 1 | 3 | 1 | 12 | 4 | 2 | 2 | 3 | 2 | 3 | 1 | 2 | 1 | 1 | 1 | 2 | 1 |

**Supplementary Table S1. *TP53* mutations in mantle cell lymphoma**

| Range(%) | MCL cells/biopsy samples(%) |
| --- | --- |
| 40-49 | 2(3.1) |
| 60-69 | 8(12.5) |
| 70-79 | 23(35.9) |
| 80-89 | 28(43.8) |
| ≥90 | 3(4.7) |
| Range(%) | p53 positive cells/MCL cells(%) |
| 0 | 42(65.6) |
| 1-9 | 7(10.9) |
| 10-29 | 9(14.1) |
| 30-49 | 3(4.7) |
| ≥50 | 3(4.7) |

**Supplementary Table S2. Percentage of MCL cells/biopsy samples and p53 positive cells/MCL cells**

|  | Sample ID | Exon | Mutation (base pair) | Mutation (amino acid) | Tv/Ts | Type | p53 |
| --- | --- | --- | --- | --- | --- | --- | --- |
| Triple positive | 1 | 6 | c.595G>A | p.G199R | Ts | Missense | ++ |
|  | 2 | 6 | c.653T>A | p.V218E | Tv | Missense | ++ |
|  | 3 | 8 | c.823T>C | p.C275R | Ts | Missense | ++ |
|  | 4 | 8 | c.845G>C | p.R282P | Tv | Missense | ++ |
| others | 5 | 5 | c.395_398 delAGAT | p.K132Sfs*37 | - | Frameshift | + |
|  | 6 | 5 | c.395A>T | p.K132M | Tv | Missense | +++ |
|  | 7 | 5 | c.423C>A | p.C141* | Tv | Nonsense | + |
|  |  | 5 | c.536A>G | p.H179R | Ts | Missense | + |
|  | 8 | 5 | c.452C>G | p.P151R | Tv | Missense | + |
|  | 9 | 7 | c.764T>A | p.I255N | Tv | Missense | +++ |
|  | 10 | 8 | c.818G>A | p.R273H | Ts | Missense | + |
|  | 11 | 9 | c.973G>A | p.G325R | Ts | Missense | - |

IHC: imunohistochemistry; -, negative; +, low positive; ++, positive; +++, high positive; Tv, transversion; Ts, transition

**Supplementary Table S3. *TP53* mutation status in triple positive patients versus the others**

| Exon | Forward Primers | Reverse primers |
| --- | --- | --- |
| 2 | 5'-CAGGTGACCCAGGGTTGGAAG-3' | 5'-GCCTGCCCTTCCAATGGATG-3 |
| 3 | 5'-GAGACCTGTGGGAAGCGAAA-3' | 5'-GCAAGGGGGACTGTAGATGG-3 |
| 4* | 5'-TGCTCTTTTCACCCATCTAC-3' | 5'-ATACGGCCAGGCATTGAAGT-3 |
| 5 | 5'-TGCTGCCGTCTTCCAGTTGCT-3' | 5'-GGGCAACCAGCCCTGTCGTC-3 |
| 6 | 5'-CGACAGGGCTGGTTGCCCAG-3' | 5'-CTCCCAGAGACCCCAGTTG-3 |
| 7 | 5'-ACAGGTCTCCCCAAGGCGCAC-3' | 5'-CAGTGTGCAGGGTGGCAAGTG-3 |
| 8 | 5'-AATGGGACAGGTAGGACCTG-3' | 5'-CTGAGGCATAACTGCACCCT-3 |
| 9* | 5'-ACAACCAGGAGCCATTGTCTT-3' | 5'-ATCTCCGCAAGAAAGGGGAG-3 |
| 10 | 5'-TGCATGTTGCTTTTGTACCGTC-3 | 5'-GGTCAGCTGCCTTTGACCAT-3 |
| 11 | 5'-CAGACCCTCTCACTCATGTG-3 | 5'-CTGACGCACAGGTATTGCAAG-3 |

*Reverse primers were used as sequencing primers, and others used forward primers as sequencing primers

**Supplementary Table S4. The sequences of PCR primers of *TP53* gene**
